# Quasi-randomization to Cannabinoid Condition in Studies of US Legal Market Cannabis: Characteristics of Accepters Versus Decliners of Condition Assignment

**DOI:** 10.1007/s11121-026-01893-4

**Published:** 2026-03-13

**Authors:** Carillon J. Skrzynski, Angela D. Bryan, Sarah J. Schmiege

**Affiliations:** 1 Department of Psychology and Neuroscience, University of Colorado Boulder, 1777 Exposition Dr, Boulder, CO 80301 USA; 2 University of Colorado Anschutz Medical Campus, 13001 E 17th Pl, Aurora, CO 80045 USA

**Keywords:** Quasi-randomization, Methodology, Health, Cannabis, THC, CBD

## Abstract

Although random assignment is the standard for drawing causal inferences within clinical trials, it is generally precluded in legal market cannabis research given its federal classification as a schedule 1 drug. Unfortunately, this may cause selection bias and compromise internal validity, and thus, alternative approaches are necessary. One such approach in this context, as well as more broadly, is quasi-random assignment whereby participants are randomly assigned to conditions but can accept or decline this assignment. This study explores whether those who accept or decline condition assignment differ in ways that impact study outcomes and informs best practices for other research areas where random assignment is not feasible or permitted. Data came from two studies examining cannabis, inflammation, and insulin sensitivity. The first included individuals who infrequently used cannabis; the second included regular users. Across studies, individuals were quasi-randomly assigned via dice roll to purchase and use either THC-dominant, CBD-dominant, or approximately equal THC:CBD ratio flower products for 1 (study 1) or 4 weeks (study 2). Demographics, cannabis use, health behaviors (e.g., exercise), and anthropometrics (i.e., body mass index [BMI]) were compared across individuals who accepted versus declined their assigned condition. Most participants accepted their assignment (83% and 63% for studies 1 and 2, respectively). Those who accepted did not differ from those who declined on any variable (*p*s > 0.11). While findings cannot rule out a selection process outside the variables assessed, results support use of this methodology in situations where true random assignment is not possible. Clinical trials: The larger project from which the current paper draws data was pre-registered on Clinicaltrials.gov (NCT04114903) on 09–06-2019.

## Introduction

While random assignment is the gold standard for establishing causal effects in research, it is not always ethical or feasible to implement (Smith & Pell, 2015). As described in classic texts on research methodology (Shadish et al., 2002), applied research projects in particular are sometimes unable or limited in their ability to randomly assign participants to conditions. In some instances, researchers are left with a comparison of conditions into which participants self-selected. Unfortunately, in such cases there is the strong potential for selection bias and thus, compromised internal validity (Shadish & Cook, 2009). A potential middle ground between random assignment and self-selection is quasi-random assignment, whereby participants are randomly assigned to a condition, but they are allowed to accept their assigned condition or decline and choose another condition. Because quasi-randomization allows for a portion of true random assignment (i.e., when some or even all participants accept their randomly assigned condition), it is a useful tool for research studies that are prevented from using true random assignment but still want to maximize internal validity.

A particular case where quasi-random assignment may be especially useful is in research on legal market cannabis products. Despite cannabis being legalized for recreational use and sold widely at commercial dispensaries at the state level, cannabis is classified as a schedule 1 drug. As such, federal regulations typically preclude clinical trials where participants are randomly assigned to use specific legal market products. Nevertheless, the legal market continues to expand the array of products available that vary in cannabinoid content and potency, specifically amount and ratio of 9-delta tetrahydrocannabinol (THC) and cannabidiol (CBD), the main constituents of cannabis. THC produces an intoxicating effect (i.e., high) associated with cannabis use, which may be appealing to some recreational users. In contrast, CBD is not intoxicating and has been associated with anxiolytic (i.e., anti-anxiety) properties, which may be more attractive for medical purposes. As such, there is growing interest in understanding both the risks and therapeutic potential of cannabis use. In this research environment, quasi-random assignment is a potential methodological option that may aid in investigating harms and benefits of legal market products as they are purchased and used in order to inform the broader public health implications of legalized cannabis.

In a recent project involving two studies of the impact of cannabis use on inflammation and insulin function (1RO1DA50515; PI: ADB), our team was denied the use of random assignment by regulatory agencies resulting in the employment of quasi-random assignment. Essentially, this entailed initially randomly assigning participants to use a THC-dominant cannabis flower product, a CBD-dominant cannabis flower product, or a cannabis flower product containing both THC and CBD. Participants rolled a die and the numbers on the die corresponded to one of these three cannabis product conditions. Participants could either accept their assignment or decline and use a product from a different condition. Importantly, studies of legal market cannabis where the participant is blind to condition are not possible, as cannabis laws require that legal market products purchased at dispensaries be accurately labeled as to the percentage and nature of cannabinoids present in the product (Kruger et al., 2022). Thus, participants must always be told what they are being asked to purchase.

Though better than no assignment at all, this strategy still presents the potential for selection effects (Shadish et al., 2002). That is, selection bias may result in groups who are substantially different from one another prior to the “intervention” of cannabis product use, and these pre-existing differences might account for any differences seen in the effects of such use. Thus, investigating what proportion of participants accept versus reject their initial assignment, and whether there are observed differences between individuals who accept their assignment versus those who do not is crucial to the interpretation of results from studies using this quasi-random assignment procedure. This question is similar in spirit to the notion of “adherers” (i.e., those who take the assigned treatment), “always-takers” (i.e., those who receive the treatment regardless of assignment [e.g., individuals who would use a certain drug, either as part of the treatment condition or as a purchased product in the control condition]), “never-takers” (i.e., those who refuse treatment no matter which condition they are assigned to), and “defiers” (i.e., those who would do the opposite of the assignment [e.g., take drug A if in group B or drug B if in group A]), categories discussed by West and Thoemmes (2010). In the case of quasi-randomization, we are essentially left with adherers (those who accept their assigned condition) and defiers (those who purposely select a different condition). Sheiner et al. (1995) would suggest that the test of interest should be limited to only the adherers to obtain a local average treatment effect (Angrist et al., 1996), also known as the complier average causal effect (Little & Yau, 1998). In the case of this study, however, the defiers still completed the study and are a potentially large and unpredictable proportion of participants given the novelty of this procedure.

As a first step in learning from this novelty, we conducted an analysis to understand whether there were systematic baseline differences between those who accepted their assigned condition (hereafter called “accepters”) versus those who rejected their assigned condition and chose a different one (hereafter called “decliners”). Identification of characteristics on which the two groups differ would suggest the strong potential for selection that would affect the interpretation of our findings. Critically, though, we can only examine characteristics which were measured, and thus even in the case of no differences between the two groups we of course cannot completely rule out selection effects (Shadish et al., 2002; VanderWeele, 2008; Vanderweele & Arah, 2011; West & Thoemmes, 2010).

The data used in these analyses came from a larger project that included two separate studies exploring the effect of cannabis use of different cannabinoid profiles on inflammation and insulin function (pre-registered on Clinicaltrials.gov: NCT04114903, registered 09–06–2019), the main findings of which have been published elsewhere and include information on procedures and participant flow (i.e., CONSORT diagram) (Bryan et al., 2025). A non-equivalent control condition of participants who did not use cannabis was included in one of the studies, but for the purposes of this paper, only the three cannabis use conditions from each study are included in analyses as only they were included in the quasi-random assignment procedure. The specific aim of the present analyses was to compare accepters to decliners on a range of demographic and other baseline characteristics potentially related to the main outcomes, including demographic information, cannabis use, alcohol use, general health behavior (e.g., exercise), and anthropometric measures (i.e., body mass index [BMI]).

The two studies, though having identical quasi-randomization procedures, focused on two different populations. Study 1 included a sample of infrequent cannabis users who had not used cannabis in at least the last 3 months and were asked to use a flower cannabis product once to assess the acute effects of cannabis use of differing cannabinoid ratios on metabolic processes. In contrast, study 2 included a sample of frequent cannabis users who were asked to switch from their regular flower product to the study cannabis flower product to which they had been assigned. They were asked to only use the study product for 4 weeks to assess more sustained influences of cannabis use of differing cannabinoid ratios on metabolic processes. Thus, both the populations (those who use cannabis infrequently versus frequently) and study procedures (using an assigned product only once versus switching from one’s regular product to an assigned product for 4 weeks) differed dramatically and could potentially affect a participant’s willingness to accept their initial condition assignment. Thus, analyses were done for each study separately.

## Study 1: Quasi-randomization Among Infrequent Cannabis Users

### Methods

#### Participants and Procedure

Participants were recruited from the greater Boulder/Denver metropolitan area via social media posts and mailed flyers, with data collection occurring between November 2019 and September 2024. Potential participants were screened for eligibility by trained research staff over the phone or through an online survey administered via REDCap, a secure data gathering system. Inclusion criteria included being 21–40 years old, stable weight (i.e., no weight fluctuations above 12 pounds in the last 6 months), and individuals must have smoked or vaped cannabis at least once since January 1, 2014, with no negative effects (e.g., paranoia, hallucinations) but not have used cannabis in the last 3 months. Individuals were not eligible if they had a known acute illness (e.g., influenza) or chronic disease (e.g., HIV, diabetes); engaged in daily tobacco use, heavy drinking (i.e., scores over 8 on the Alcohol Use Disorder Identification Task [AUDIT; Saunders et al., 1993]), or illicit drug use; currently used medications for anti-inflammation, immunosuppression, or glucose lowering; had a current or past history of psychotic or bipolar disorder or used anti-psychotics or other medications to treat bipolar disorder; had donated blood in the 8 weeks prior to screening or intended to donate in the 8 weeks after study completion; had a fasting blood glucose outside of 55 and 126 mg/dL at the baseline appointment; were on a carbohydrate restricted diet; or were pregnant, nursing, or trying to become pregnant (where applicable).

#### Procedures

Study 1 included a baseline appointment and an appointment which occurred in our mobile pharmacology laboratory 1 week following the baseline session. The current analysis includes data from the baseline session only. During this appointment, participants provided informed consent and completed surveys in an electronic format using REDCap (see “Measures”). After this, they were quasi-randomly assigned to purchase and use a particular cannabis flower product via dice roll. As mentioned earlier, participants rolled a die with two of the six numbers corresponding to one of three product use conditions: cannabis flower product that was either THC-dominant (0% CBD, 23% THC), CBD-dominant (20% CBD, 1% THC), or contained approximately equal parts THC and CBD (8% CBD, 10% THC). After rolling, participants were told which product condition they were assigned to but could either accept that assignment or choose another.^1^

The study and all materials were approved by the University of Colorado Institutional Review Board. Analyses for the current paper are exploratory and were not pre-registered. All data, analysis code, and research materials are available by request to the corresponding author.

#### Measures

##### Demographics

Participants self-reported information including age, gender,^2^ and race.

##### Cannabis Use

Total days of cannabis use in the last month was measured via a version of the 30-day Timeline Followback (TLFB; Sobell & Sobell, 1992) modified for online use (O-TLFB; Martin-Willett et al., 2019).

##### Alcohol Use

Past year hazardous alcohol use was measured via the Alcohol Use Disorder Identification Task (AUDIT; Saunders et al., 1993). Items for this measure are summed, and higher scores indicate more hazardous drinking.

##### Sleep

The Pittsburgh Sleep Quality Index (PSQI; Buysse et al., 1989) was used to assess sleep quality over the past month. Higher scores indicate worse sleep, with a global score of 5 or above indicating poor sleep quality.

##### Diet

Participants reported on their diet quality via the Nutrition Data System for Research (NDSR; *Nutrition Data System for Research,* (n.d.) software versions 2019, 2020, and 2021, developed by the Nutrition Coordinating Center (NCC), University of Minnesota, Minneapolis, MN. Information from the NDSR version 2022 was used to calculate Healthy Eating Index (HEI) scores with scores above 80 considered a good diet and below 80 in need of improvement.

##### Exercise

Trained research staff administered the Stanford 7-day Physical Activity Recall (PAR; Blair et al., 1985) where participants reported their total days of exercise, minutes of moderate exercise, minutes of hard exercise, and minutes of very hard exercise during the past 7 days.

##### Anthropomorphic Assessments

BMI was calculated using height and weight measured with a digital scale and height rod (Tanita WB-800H; Tanita Corporation, Tokyo, Japan).

##### Cannabis attitudes, expectancies, and identity

Participants completed a five-item survey on attitudes toward smoking marijuana (Ito et al., 2015). An example item is “For me, using cannabis regularly would be…” with response options from 1, Bad to 7, Good (Cronbach’s *α* = 0.87).

Participants additionally completed a modified version of the Marijuana Effect Expectancy Questionnaire — Brief (MEEQ-B; Torrealday et al., 2008) which included four additional items related to the aims of the broader project (i.e., “marijuana makes people… eat unhealthy food; drink less alcohol; less motivated to exercise; get better sleep” included as positive or negative expectancies as appropriate). Response options ranged from 1, Strongly Disagree to 5, Strongly Agree (Cronbach’s *α*s = 0.60 for both positive and negative expectancy scales).

Finally, participants completed five items assessing identification with cannabis as part of one’s personality or identity (Blevins et al., 2018) (e.g., “Smoking marijuana is part of my self-image” from 1, Strongly Disagree to 10, Strongly Agree, Cronbach’s *α* = 0.85). Higher scores indicated greater endorsement in all cases (i.e., more positive cannabis attitudes, greater identification with cannabis as part of one’s identity, and more negative/positive expectancies).

### Data Analysis

Posit Team, (2025) was used for all analyses. First, data were examined to determine normality across all variables via skew and kurtosis. In cases where skew and kurtosis were greater than conventional thresholds (i.e., ± 2 for skew and ± 7 for kurtosis), potential outliers were examined. Findings demonstrated that there were four instances for cannabis identity, three for minutes of moderate PA, and six for minutes of very hard PA. In these cases, datapoints were winsorized to 3 standard deviations (SDs) above the mean for each variable. Following this, all variables were compared across accepters and decliners via *t*-tests or *χ*^2 ^tests depending on whether the variable was continuous or categorical, respectively.

## Results

**Fig. 1:**
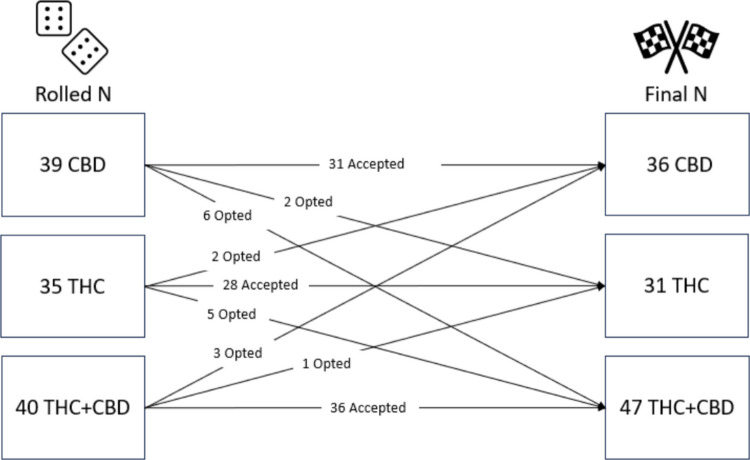
Changes from original die roll assignment to final assignment in study 1

The majority of study 1 participants (*N* = 114, *M*_age_ = 29.87, 57% female, and 88% white) accepted their condition assignment (83%; *N* = 95) while 17% (*N* = 19) declined their assignment and switched to a different product group. Originally, 39 individuals rolled the CBD condition while 35 rolled the THC condition and 40 rolled the THC + CBD condition but the final *N*s were 36, 31, and 47, respectively (see Fig. 1).

*T*-tests and *χ*^2^tests did not reveal any differences between accepters and decliners on any variable of interest (see Table 1). Both groups were majority white, were composed of more female than male participants, and were, on average, about 30 years old. Consistent with recruitment criteria, individuals reported engaging in very infrequent cannabis use with less than 1 day of use in the last month across both groups. Cannabis use attitudes and identity were comparably low (i.e., attitudes were slightly negative, and individuals generally did not see cannabis as part of their identity) while the average positive and negative cannabis use expectancies were slightly higher than neutral and neutral, respectively (i.e., average was closer to 4, Somewhat agree and close to 3, Uncertain, respectively). Finally, both groups reported similarly high amounts of minutes of moderate, hard, and very hard exercise but comparably poor diet and sleep quality. Both groups had a mean BMI around the normal weight range and did not demonstrate hazardous alcohol use.

**Table 1:** Descriptive information across decliners and accepters in study 1

	Accepters (*N* = 95)	Decliners (*N* = 19)	Cohen’s *d*^e^	*p*-values
Female (*n* (%))	53 (55.79)	12 (63.16)	0.00	0.74
White (*n* (%))	85 (89.47)	15 (78.95)	0.15	0.37
Age (*M* (SD))	29.36 (5.28)	29.79 (6.21)	− 0.08	0.78
Cannabis identity (*M* (SD))	1.12 (0.37)	1.06 (0.12)	0.18	0.19
Positive cannabis expectancies (*M* (SD))	3.80 (0.49)	3.74 (0.58)	0.13	0.64
Negative cannabis expectancies (*M* (SD))	3.36 (0.6)	3.40 (0.72)	− 0.06	0.82
Cannabis attitudes (*M* (SD))	2.90 (1.11)	2.84 (1.06)	0.05	0.84
Cannabis use days/month (*M* (SD))	0.05 (0.3)	0.11 (0.32)	− 0.17	0.51
Total HEI^a^ score (*M* (SD))	55.03 (15.72)	55.91 (13.62)	− 0.06	0.80
AUDIT^b^ score (*M* (SD))	3.25 (2.64)	3.16 (2.81)	0.04	0.89
PSQI^c^ score (*M* (SD))	5.01 (3.25)	5.00 (3.25)	0.00	0.99
Mod min (*M* (SD))	239.75 (230.85)	288.32 (324.38)	− 0.20	0.54
Hard min (*M* (SD))	111.24 (147.35)	132.89 (144.6)	− 0.15	0.56
Very hard min (*M* (SD))	43.77 (88.43)	49.65 (111.66)	− 0.06	0.83
BMI^d^ (*M* (SD))	24.87 (4.46)	23.77 (3.28)	0.26	0.22

^a^*HEI*, Healthy Eating Index

^b^*AUDIT*, Alcohol Use Disorder Identification Task

^c^*PSQI*, Pittsburgh Sleep Quality Index

^d^*BMI*, body mass index

^e^Cohen’s *d* for categorical variables was generated by first calculating the phi coefficient and then converting using the formula 2 × *φ* × sqrt(1 − *φ*^2^)

## Study 2: Quasi-randomization Among Regular Cannabis Users

### Methods

#### Participants and Procedure

Recruitment and screening followed the same procedure as for study 1 but occurred from June 2020 to July 2022. Eligibility requirements were the same as for study 1 with the exception that individuals in this study had to engage in regular (at least weekly) cannabis use for at least a year. Study 2 participants engaged in a baseline session and a follow-up session 4 weeks later with daily survey data collected in between; however, like in study 1, only baseline data was used for the current analyses.

#### Measures

Measures were the same as in study 1, including collection of information on demographics, alcohol use, sleep, diet, exercise, and cannabis attitudes, expectancies, and identity.^3^ However, because individuals regularly used cannabis, additional cannabis use assessments were included. This included total days of cannabis use as described above as well as the number of times cannabis was used per day (1 = “1 time per day” to 6 = “6 or more times per day”), average grams used at one time, and average THC and CBD percent of typically used products.

### Data Analysis

All analytic procedures were the same as in study 1. When examining outlying data, there were three instances of outlying data for number of cannabis grams used daily, two instances for BMI, three for minutes of moderate PA, two for minutes of hard PA, and three for minutes of very hard PA. Like with study 1, datapoints were winsorized to 3 SDs above the mean for each variable and then variables were compared across accepters and decliners via *t*-tests and χ2 tests.

## Results

**Fig. 2:**
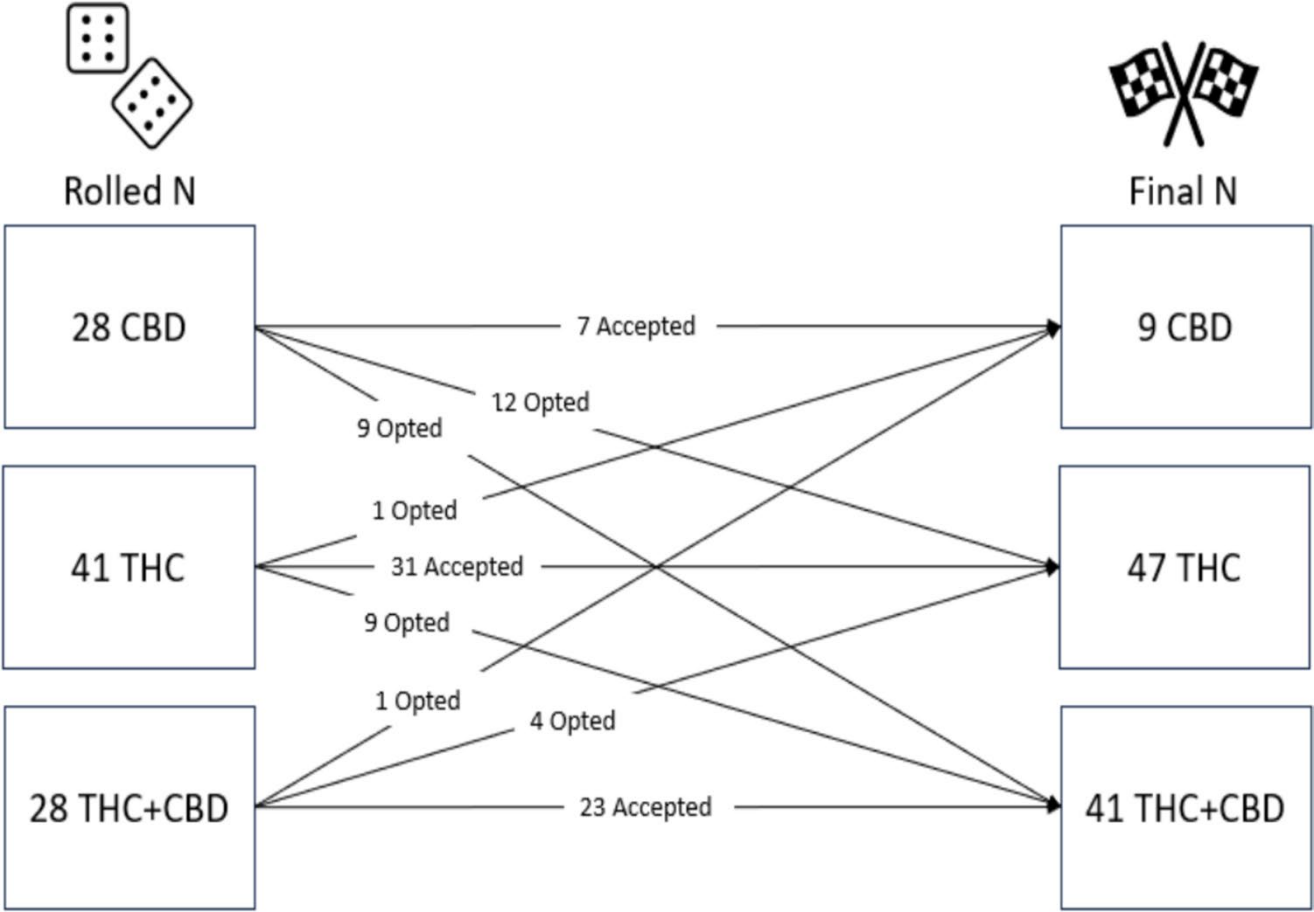
Changes from original die roll assignment to final assignment in study 2

The majority of study 2 participants (*N* = 97, *M*_age_ = 29.87, 35% female, and 77% white) accepted their condition assignment (63%; *N* = 61), while 37% (*N* = 36) declined their assignment and switched to a different product group. Originally, 28 individuals rolled the CBD condition, 41 rolled the THC condition, and 28 rolled the THC + CBD condition. However, several individuals opted out resulting in only 9 individuals in the CBD condition (75% opted out), with 47 and 41 in the THC and THC + CBD conditions, respectively (see Fig. 2). Participants in study 2 were significantly more likely to change their condition assignment than individuals in study 1 (*χ*^2^ = 11.37, *p* < 0.001).

Like in study 1, *t*-tests and *χ*^2^ tests did not reveal any differences between accepters and decliners on any variable of interest (see Table 2). Again, both groups were majority white and were, on average, about 30 years old. However, in contrast to study 1, study 2 had a significantly greater composition of male participants (*χ*^2^ = 8.39, *p* < 0.01).

**Table 2:** Descriptive information across decliners and accepters in study 2

	Accepters (*N* = 61)	Decliners (*N* = 36)	Cohen’s *d*^e^	*p*-value
Female (*n* (%))	24 (39.34)	11 (30.56)	0.00	0.30
White (*n* (%))	55 (90.16)	29 (80.56)	0.18	0.51
Age (*M* (SD))	29.59 (4.92)	30.33 (5.23)	− 0.15	0.49
Cannabis use days (*M* (SD))	21.54 (7.46)	22.06 (7.82)	− 0.07	0.75
Daily frequency (*M* (SD))	2.57 (1.63)	2.50 (1.75)	0.04	0.84
Average grams per occasion (*M* (SD))	0.16 (0.06)	0.21 (0.21)	− 0.38	0.16
Average THC percent (*M* (SD))	18.69 (5.24)	19.66 (6.94)	− 0.17	0.53
Average CBD percent (*M* (SD))	4.40 (6.02)	4.85 (6.68)	− 0.07	0.79
Cannabis identity (*M* (SD))	3.93 (2.31)	3.45 (1.98)	0.22	0.28
Positive cannabis expectancies (*M* (SD))	3.95 (0.61)	4.10 (0.45)	− 0.26	0.18
Negative cannabis expectancies (*M* (SD))	2.96 (0.66)	2.78 (0.72)	0.26	0.24
Cannabis attitudes (*M* (SD))	5.15 (1.26)	5.26 (1.14)	− 0.08	0.69
Total HEI^a^ score (*M* (SD))	51.03 (15.37)	48.70 (13.36)	0.16	0.44
AUDIT^b^ score (*M* (SD))	3.92 (2.53)	3.58 (3.00)	0.12	0.58
PSQI^c^ score (*M* (SD))	5.60 (3.14)	5.42 (3.06)	0.06	0.80
Mod min (*M* (SD))	350.22 (419.33)	345.56 (430.97)	0.01	0.96
Hard nin (*M* (SD))	104.23 (182.65)	129.89 (219.24)	− 0.13	0.56
Very hard min (*M* (SD))	31.68 (86.82)	19.72 (49.48)	0.16	0.39
BMI^d^ (*M* (SD))	23.75 (4.31)	25.26 (4.57)	− 0.34	0.11

^a^*HEI*, Healthy Eating Index

^b^*AUDIT*, Alcohol Use Disorder Identification Task

^c^*PSQI*, Pittsburgh Sleep Quality Index

^d^*BMI*, Body Mass Index

^e^Cohen’s *d* for categorical variables was generated by first calculating the phi coefficient and then converting using the formula 2 × *φ* × sqrt(1 − *φ*^2^)

Across accepters and decliners, participants engaged in an average of approximately 22 total days of cannabis use in the past month, used approximately 2.5 times per day, and an average of about 0.20 g. Interestingly, like with study 1, participants indicated low cannabis identity and attitudes scores as well as slightly positive (i.e., average close to 4, Somewhat agree) and neutral (i.e., average close to 3, uncertain) positive and negative cannabis use expectancies, respectively. Similarly, both groups engaged in high amounts of very hard, hard, and moderate exercise use and demonstrated poor diet and sleep quality. Finally, accepters and decliners engaged in low levels of hazardous alcohol use while the average BMI for accepters was in the normal range and only slightly above normal in the decliner group; a difference that was not statistically significant.

## Discussion

This analysis examined the use of a quasi-randomization procedure to assign individuals from two separate studies to specific cannabis use products for study use. Findings indicated that across both studies, most participants accepted their assignment. Additionally, accepting versus declining was not associated with any substance use pattern, health behavior, or measure of cannabis attitude, expectancy, or identity in either study. These findings are consistent with other studies that have compared groups of participants who did versus did not accept random assignment to condition and found them to be largely similar (e.g., Juster et al., 1995).

Despite these encouraging findings, some interesting differences did emerge across the studies. Chief among them is that acceptance was significantly higher among study 1 participants compared to study 2 participants. This is not necessarily surprising, though, as individuals in study 1 were infrequent cannabis users and may not have had specific preferences for cannabinoid content in the same way that individuals in study 2, who used more regularly, might. Importantly, participants in study 1 were only asked to use their study product once, while participants in study 2 were asked to switch from using their preferred product to a study product exclusively for the course of 4 weeks. Given these study differences, the fact that study 2 participants declined more often than study 1 participants, and preferentially declined use of the CBD-dominant product, is not unexpected. Indeed, the average percent of THC in products typically used by participants in study 2 was around 19% as compared to 4.5% for CBD content suggesting that study 2 participants preferred products with higher potency of THC to achieve desirable intoxicating effects (i.e., high) relative to CBD-dominant products that do not produce intoxicating effects. Overall, it was perhaps a bigger “ask” for participants in study 2 to drastically change the product they were used to consuming on a regular basis, and to do so for an extended period of time (i.e., 4 weeks).

The present findings should be considered in light of study limitations. One limitation is that across the studies, the samples were majority white, which may hinder generalizability to more diverse populations. Future work with greater inclusion of a wider variety of racial backgrounds is indicated. A larger limitation is the small sample size for those who declined, especially within study 1 (*N* = 19 out of 114). Because of this, it is possible that we were underpowered to detect differences across accepters and decliners, especially sex differences which appeared disproportional across groups. Indeed, Cohen’s *d* would have needed to be 0.65 for us to detect a statistically significant difference between groups. Of course, this is based on a potentially imprecise estimate given the skewed distribution of decliners to acceptors. Despite this, the overall small differences seen across multiple outcomes within both studies suggest that individuals who accepted versus declined cannabis condition assignment were similar in all characteristics studied and support the feasibility of our quasi-randomization method.

Some methodological aspects are worth noting, however. In the second study, the utilization of a participant dice roll (necessary so that study staff was not randomly assigning participants to condition) resulted purely by chance in “faulty randomization” (Shadish et al., 2002), in that many fewer dice rolls corresponded to the CBD-dominant product condition. As such, different approaches might be employed in future studies that allow participants agency in “choosing” their random condition while also being more likely to equalize across groups. For example, one tactic might be having participants draw from a bucket containing a number of slips of paper equal to the full sample size to be recruited. The slips of paper would have designations corresponding to conditions and would appear in equal amounts of slips per condition. This would ensure both randomness and equal numbers of initial assignments per condition. Another methodological change that might be useful in the context of this study would be to allow participants to use other products in addition to their assigned product (i.e., ask participants to add a new product in addition to what they regularly use). In a perfect world, quasi-randomization becomes total random assignment when there are no decliners, so the goal to minimize declining of random assignment might be well served with this option.

It is worth noting, however, that even in traditional random clinical trials (RCTs) that employ random assignment, there is still the possibility that some individuals will not adhere to their assigned condition (Shadish et al., 2002). As such, regardless of the assignment mechanism, it is important to track and appropriately address instances where condition assignment does not match condition participation. With that in mind, another option to pursue is the use of different methods of analysis that seek to correct for unmeasured sources of bias (Carnegie et al., 2016; Streeter et al., 2017). In the case of non-random assignment, Streeter et al. (2017) reviewed the literature of studies that corrected for unmeasured confounding and found that an instrumental variable approach was the most common. Instrumental variable analyses do, however, typically require substantially larger sample sizes than were available in this study (Wang et al., 2018). Another increasingly used option for studies where randomization is not possible is propensity score matching; however, this method is not appropriate for situations with “partial treatment implementation” (Rindskopf et al., 2018, pg. 256) as occurred in this study.

In the case of studies utilizing some form of randomization, analyses can be implemented that consider the inclusion of decliners into the group matching the product they actually used (i.e., per protocol in RCTs) versus into the group matching the product that they were *originally assigned* to use (i.e., intent-to-treat (ITT) approach in RCTs; Lachin, 2000). There are certainly arguments favoring both approaches (Fisher, 2017). A per-protocol approach gives an estimate of the efficacy of the treatment among those who received it while the ITT approach gives an estimate of the effectiveness of the treatment as it would be prescribed in the clinic.

In the context of the larger project for which these analyses are most relevant, the current situation is not a perfect match to either of these cases as we are not testing the “efficacy of a treatment” per se nor do we have uncertainty about which condition participants actually followed as is the case when participants are lost to follow-up. Nevertheless, we favor the perspective of Sheiner et al. (1995) that in this case the effect of the cannabis product as used is more relevant to our research question, in that as the cannabis landscape currently stands patients do not receive prescriptions for legal market cannabis products. As such, the planned primary method for main analyses followed a per-protocol approach, categorizing participants’ condition based on the actual product they used. However, a secondary analysis using an ITT approach as well as a sensitivity analysis including only accepters can be conducted in future analyses to facilitate confidence in the interpretation of our findings.

Methodological/analytic approaches and limitations aside, the findings provide evidence that the quasi-randomization procedure was not associated with systematic differences on any measure we examined between those who accepted versus declined their assignment. Participants found the dice roll procedure understandable and most accepted their condition assignment. While the possibility remains that other variables not assessed might influence the selection process in these studies, the data indicate that quasi-random assignment like that used here is a viable option for situations in which true random assignment cannot be exercised. Though the current study utilized this paradigm in the context of cannabis use, to the extent that quasi-randomization is available, given that it approximates random assignment, it may also be a useful tool in other areas (e.g., educational settings or other public health domains).

## Data Availability

All data, analysis code, and research materials are available by request to the corresponding author.

## Footnotes

1 While participants told us whether or not they accepted their condition, categorization to the accepter versus decliner groups was done based on the actual product that participants purchased and used which was verified through a REDCap survey participants completed after the baseline appointment including a picture of the product label. In cases where the product was different than the original assignment, participants were classified as “decliners” while if it adhered to the assignment, they were classified as “accepters”.

2 In two cases, individuals identified as “other” and sex at birth was used to remove the inclusion of a separate, underpowered category in analyses.

3 Reliability for these last three measures were comparable to in study 1.
